# Activating transcription factor 3 regulates hepatic apolipoprotein A4 upon metabolic stress

**DOI:** 10.1016/j.jbc.2025.108468

**Published:** 2025-03-28

**Authors:** Jasmine Encarnacion, Danielle M. Smith, Joseph Choi, Joseph Scafidi, Michael J. Wolfgang

**Affiliations:** 1 Department of Physiology, The Johns Hopkins University School of Medicine, Baltimore, Maryland, USA; 2 Department of Pharmacology and Molecular Sciences, The Johns Hopkins University School of Medicine, Baltimore, Maryland, USA; 3 Department of Biological Chemistry, The Johns Hopkins University School of Medicine, Baltimore, Maryland, USA; 4 Department of Neurology, The Johns Hopkins University School of Medicine, Baltimore, Maryland, USA; 5 The Michael V. Johnston Center for Developmental Neuroscience, Kennedy Krieger Institute, Baltimore, Maryland, USA

**Keywords:** apolipoprotein A4, Atf3, fatty acid oxidation, gene knockout, liver metabolism, metabolic regulation, sex-dependent, transcription factor

## Abstract

The liver plays essential roles in maintaining systemic glucolipid homeostasis under ever changing metabolic stressors. Metabolic dysregulation can lead to both adaptive and maladaptive changes that impact systemic physiology. Here, we examined disparate genetic and environmental metabolic stressors and identified apolipoprotein A4 (ApoA4) as a circulating protein upregulated in liver-specific KOs for carnitine palmitoyltransferase 2 and pyruvate carboxylase. We found this upregulation to be exacerbated by fasting and high-fat or ketogenic diets. Unique among these models was a concomitant increase in activating transcription factor 3 (Atf3). Liver-specific overexpression of Atf3 resulted in increased ApoA4 expression in a sex-dependent manner. To understand the requirement of Atf3 to metabolic stress, we generated liver-specific Atf3, Cpt2 double KO mice. These experiments demonstrated the requirement for Atf3 in the induction of ApoA4 mRNA, ApoA4 protein, and serum triglycerides that were also sex-dependent. These experiments reveal the roles of hepatic Atf3 and ApoA4 in response to metabolic stress *in vivo*.

The liver coordinates systemic metabolism in response to dietary state by maintaining the homeostasis of circulating metabolites. Regarding lipid homeostasis, the metabolic flexibility of hepatocytes allows the liver to shift between fatty acid synthesis and beta-oxidation as necessary, contributing to the storage of triglycerides or the production of ketones during fed and fasted states, respectively (1, 2). Conditions that impede these activities, including genetic deficiencies of metabolic pathways, result in hepatic stress and a myriad of problems associated with either the lack of sufficient substrate or the inappropriate accumulation of toxic compounds (3). While there is abundant research surrounding the biochemical regulation of hepatic metabolism, the complexity of these pathways leaves many of its intricacies unknown. To emulate relevant genetic deficiencies and further examine the role of their respective metabolic enzymes, we previously developed several models of hepatic metabolic dysfunction. Using the Cre/Lox system, we have generated liver-specific KOs for carnitine palmitoyltransferase 2 (Cpt2^L−/−^) (4, 5), pyruvate carboxylase (PCx^L−/−^) (6), pyruvate dehydrogenase E1a, tuberous sclerosis complex 1, and the insulin receptor.

In this study, we found apolipoprotein A4 (ApoA4) upregulation in a serum protein screen for common signals of metabolic stress across mouse models. This apolipoprotein, like others in the family, is involved in lipid transport through the serum; ApoA4 can be found on chylomicrons or HDL particles prior to release into the plasma (7). ApoA4 is primarily expressed in the postprandial state from the small intestine; however, the liver is also a contributor of serum ApoA4 particularly in rodents, with a documented role in human liver as well (8). In addition to intestinal lipid metabolism, ApoA4 has been shown to play roles in food intake and glucose homeostasis and even plays a role in restricting hepatic steatosis (8, 9, 10). Wang *et al.* showed aggravated hepatic steatosis in ApoA4 KO rats when triggered by a 16-h fast (11), suggesting an important role of ApoA4 in lipid export from the liver upon fasting. The data presented here demonstrating upregulated ApoA4 in fasted mouse models not only support the role of ApoA4 in maintaining hepatic lipid homeostasis but also point to a distinct regulatory pathway for hepatic ApoA4 expression under conditions of metabolic stress.

While several proteins that influence ApoA4 expression have been identified, little is known about the regulatory distinctions between gut and liver ApoA4 production. The CREB/activating transcription factor (ATF) family is involved in many aspects of liver metabolism, with its dysregulation being linked to metabolic diseases such as metabolic dysfunction–associated fatty liver disease (12). Residing in this family are documented ApoA4 transcriptional regulators LUMAN and CREBH, encoded by genes CREB3 and CREB3L3, respectively (13, 14).

Here, we identify another member of the CREB/ATF family, Atf3, as a player in the upregulation of ApoA4 within models of hepatic metabolic dysfunction. In previous experiments, we showed Atf3 mRNA to be upregulated in the livers of several disparate models of enzyme deficiency following a fast, concomitant with the upregulation of ApoA4 we observe here (4, 5, 6, 15). As a stress-inducible gene, Atf3 is linked to a wide range of systemic metabolic activities from glucose metabolism to immunity to oncogenesis (16). Regarding liver metabolism: increased Atf3 expression has been associated with hepatic steatosis and impaired glucose homeostasis in ZDF rats (17) and Atf3-overexpressing mice (18, 19), but other studies exhibit protective effects of liver-specific Atf3 on hepatic lipid metabolism and atherosclerosis in both human and mouse models of obesity and metabolic dysfunction–associated fatty liver disease (19, 20, 21). Like ApoA4, Atf3 is also closely involved with reverse cholesterol transport *via* the uptake of HDL into the liver (21, 22). Although a direct link between Atf3 activity and ApoA4 transcription is yet to be established, Xu *et al.* reported a robust increase in hepatic ApoA4 mRNA in mice upon liver-specific overexpression of human Atf3 (21). Here, we investigate the connection between hepatic Atf3 and ApoA4 regulation under varying conditions of metabolic dysfunction and discover a sexually dimorphic impact of Atf3 on the expression and activity of ApoA4.

## Results

### Disparate models of metabolic stress increase ApoA4 levels in serum

**Figure 1:**
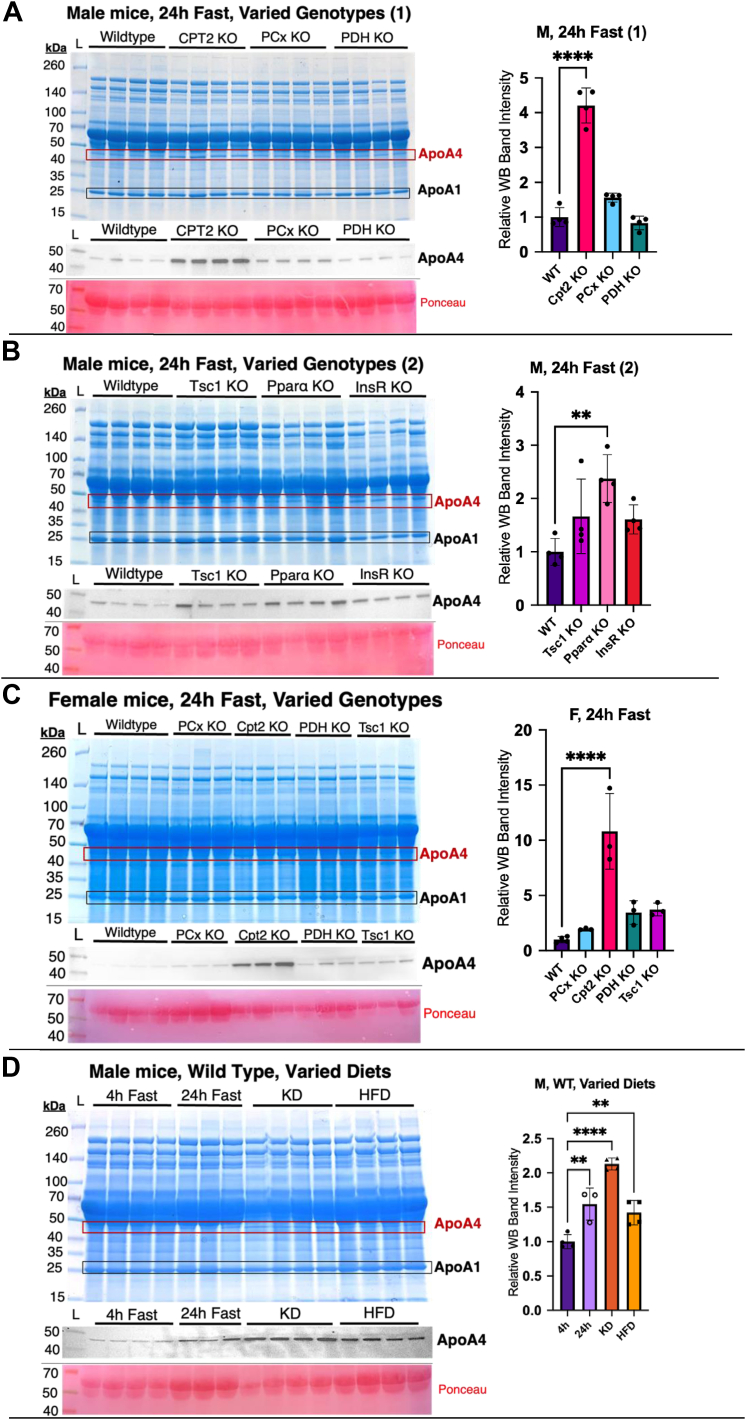
**Serum protein screening shows ApoA4 upregulation in several models of dysregulated hepatic metabolism.** Coomassie-stained SDS-PAGE gels of serum proteins, followed by Western blots for ApoA4 with ponceau stained albumin bands for loading control. *A*, serum was derived from male mice, fasted 24 h, with WT, Cpt2^L−/−^, PCx^L−/−^, PDH^L−/−^ genotypes. n = 4. *B*, comparison of WT, TSC1^L−/−^, Pparα^−/−^, and InsR^L−/−^ genotypes; n = 4. *C*, serum derived from female mice, fasted 24 h, with WT, PCx^L−/−^, Cpt2^L−/−^, PDH^L−/−^, and TSC1^L−/−^ genotypes; n = 3. *D*, serum derived from WT male mice in various dietary states: Chow-fed 4 h fast, chow-fed 24 h fast, high-fat diet–fed 4 h fast, and ketogenic diet–fed 4 h fast; n = 4. Relative values for Western blot band intensities are illustrated. Statistical significance was determined by ordinary one-way ANOVA with the Brown-Forsythe test, Bartlett's test, and Bonferroni multiple comparisons test, conducted with GraphPad prism; n = 4. Data expressed as mean ± SD. ∗*p* < 0.05, ∗∗*p* < 0.01, ∗∗∗*p* < 0.001, and ∗∗∗∗*p* < 0.0001. ApoA4, apolipoprotein A4; Cpt2, carnitine palmitoyltransferase 2; PCx, pyruvate carboxylase; InsR, insulin receptor; PDH, pyruvate dehydrogenase; TSC1, tuberous sclerosis complex 1.

The liver affects systemic metabolism *via* endocrine or bulk transport through proteins secreted through the circulation. To better understand how metabolic stress impacts the serum proteome and therefore systemic physiology, we screened a series of liver specific KO mouse models with perturbations in macronutrient metabolism or cell signaling for changes in serum proteome (4, 5, 6, 15). Examining Coomassie blue-stained SDS-PAGE gels loaded with 24-h-fasted mouse serum revealed a strong upregulation in a 46 kDa band for a subset of KO models compared to their WT counterparts with floxed alleles (Fig. 1). Mass spectrometry revealed this band as ApoA4, and a separate 28 kDa band that did not appear to vary across models as ApoA1 (23). Western blotting confirmed the 46 kDa protein as ApoA4.

We quantified the Western blot bands and found significant ApoA4 upregulation in the serum of Cpt2 liver-specific (Fig. 1*A*) and Pparα whole body (Fig. 1*B*) KOs suggesting that deficits in hepatic fatty acid catabolism were accompanied by an increase in circulating ApoA4 in the fasted state. No changes were observed in pyruvate dehydrogenase E1a, tuberous sclerosis complex 1, or insulin receptor liver-specific KO mice. We also observed a trend toward elevated ApoA4 levels in liver-specific PCx KOs in the fasted state that did not reach statistical significance. (Fig. 1*A*). These changes in circulating ApoA4 were observed in female mice as well (Fig. 1*C*). Quantifying the intensity of the female Western blot bands demonstrated a significant increase in serum ApoA4 for Cpt2^L−/−^ mice, confirming a loss in hepatic fatty acid oxidation to have the greatest serum ApoA4 increase of those assayed.

Given the robust differences in circulating ApoA4 in mice with a loss in hepatic fatty acid oxidation, we questioned whether dietary lipids could affect ApoA4 levels. We examined the serum proteins of WT mice fed high-fat diet (HFD) or ketogenic diet (KD), which can both induce increased hepatic triglyceride content when administered to healthy mice (24, 25). We assessed the serum of mice fed a standard chow diet fasted for 4 h, chow-fed mice fasted for 24 h, and mice fed matched KD or HFD for changes in serum ApoA4 levels (6). The quantified Western blot showed a significant increase of ApoA4 protein in 24-h-fasted, KD, and HFD mouse serum compared to chow fed mice, with the greatest increase in mice administered KD (Fig. 1*D*). These data show an upregulation of ApoA4 under various metabolic and nutritional states that increase hepatic lipid burden, with the KD producing the most dramatic phenotype followed by a 24 h fast.

### Deficiencies in PCx or fatty acid oxidation potentiate the effect of diet on serum ApoA4

**Figure 2:**
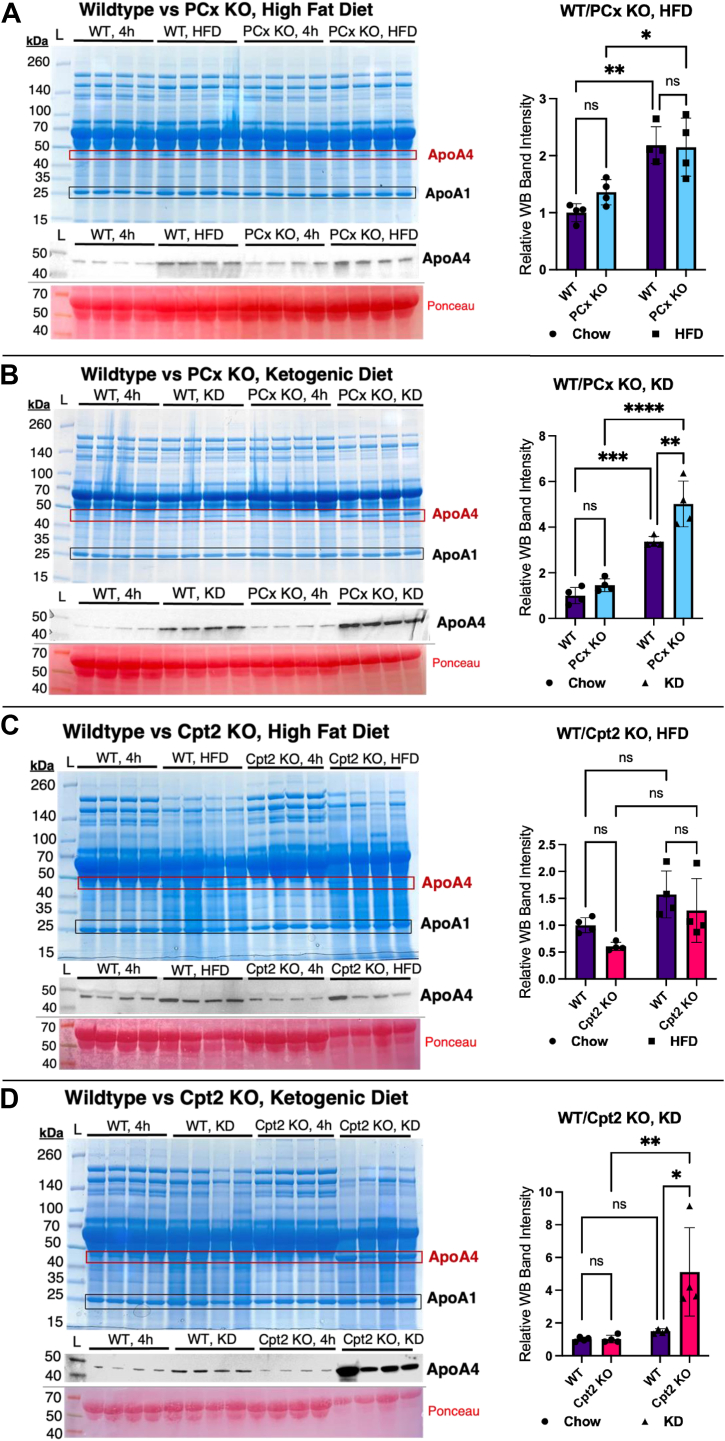
**Changes in serum ApoA4 levels vary across PCx^L−/−^ and Cpt2^L−/−^ mice fed chow, high-fat, and ketogenic diets.** Coomassie-stained SDS-PAGE gel of serum proteins, followed by Western blots for ApoA4 with ponceau-stained albumin bands for loading control. Serum was collected from male mice, fasted 4 h, in the following groups (n = 4): (*A*) PCx^L f/f^ (phenotypically WT) on chow or HFD, PCx^L−/−^ (liver knockout) on chow or HFD; (*B*) PCx^L f/f^ on chow or KD, PCx^L−/−^ on chow or KD; (*C*) Cpt2^L f/f^ on chow or HFD, Cpt2^L−/−^ on chow or HFD; (*D*) Cpt2^L f/f^ on chow or KD, and Cpt2^L−/−^ on chow or KD. Relative values for Western blot band intensities are illustrated. Statistical significance was determined by two-way ANOVA with Tukey’s multiple comparisons test, conducted with GraphPad prism; n = 4. Data expressed as mean ± SD. ∗*p* < 0.05, ∗∗*p* < 0.01, ∗∗∗*p* < 0.001, and ∗∗∗∗*p* < 0.0001. ApoA4, apolipoprotein A4; Cpt2, carnitine palmitoyltransferase 2; HFD, high-fat diet; KD, ketogenic diet; PCx, pyruvate carboxylase.

To determine whether combining models of metabolic stress would potentiate serum ApoA4 levels, we assayed serum in PCx^L−/−^ and Cpt2^L−/−^ mice on KD and HFD (4, 5, 6). Compared to their WT counterparts, we observe an amplification of the diet-induced ApoA4 upregulation for both PCx and Cpt2 liver KO mice (Fig. 2). On an HFD, the serum ApoA4 band was more pronounced in PCx^L−/−^ mice compared to chow diet, but there was no significant difference between HFD WTs and liver KOs (Fig. 2*A*). On the other hand, PCx^L−/−^ mice fed a KD diet showed higher ApoA4 than WT littermates, far exceeding the effect of KD alone (Fig. 2*B*). Similarly, the Cpt2^L−/−^ mice showed a striking increase in serum ApoA4 when fed a KD compared to their WT littermates (Fig. 2*D*), while HFD did not produce any significant differences (Fig. 2*C*).

We previously demonstrated extreme metabolic stress followed by lethality in Cpt2 and PCx liver KO mice fed a KD (4, 6). Despite the differences between the activity of these enzymes and their impact on lipid metabolism, both PCx and Cpt2 LKO mice experienced rapid weight loss and death at around the 1-week mark. Importantly, an HFD did not produce the same metabolic stress. Here, although HFD produced slightly elevated ApoA4 levels in both control and PCx^L−/−^ mice, there was no significant difference of serum ApoA4 in either PCx or Cpt2 KOs compared to their floxed counterparts fed HFD. The distinction between the KD and HFD response suggests a distinct stress pathway triggered where hepatic fatty acid oxidation is required as the primary source of fuel but is enzymatically restricted.

### Hepatic ApoA4 is dramatically overexpressed in response to metabolic stress compared to other apolipoproteins

**Figure 3:**
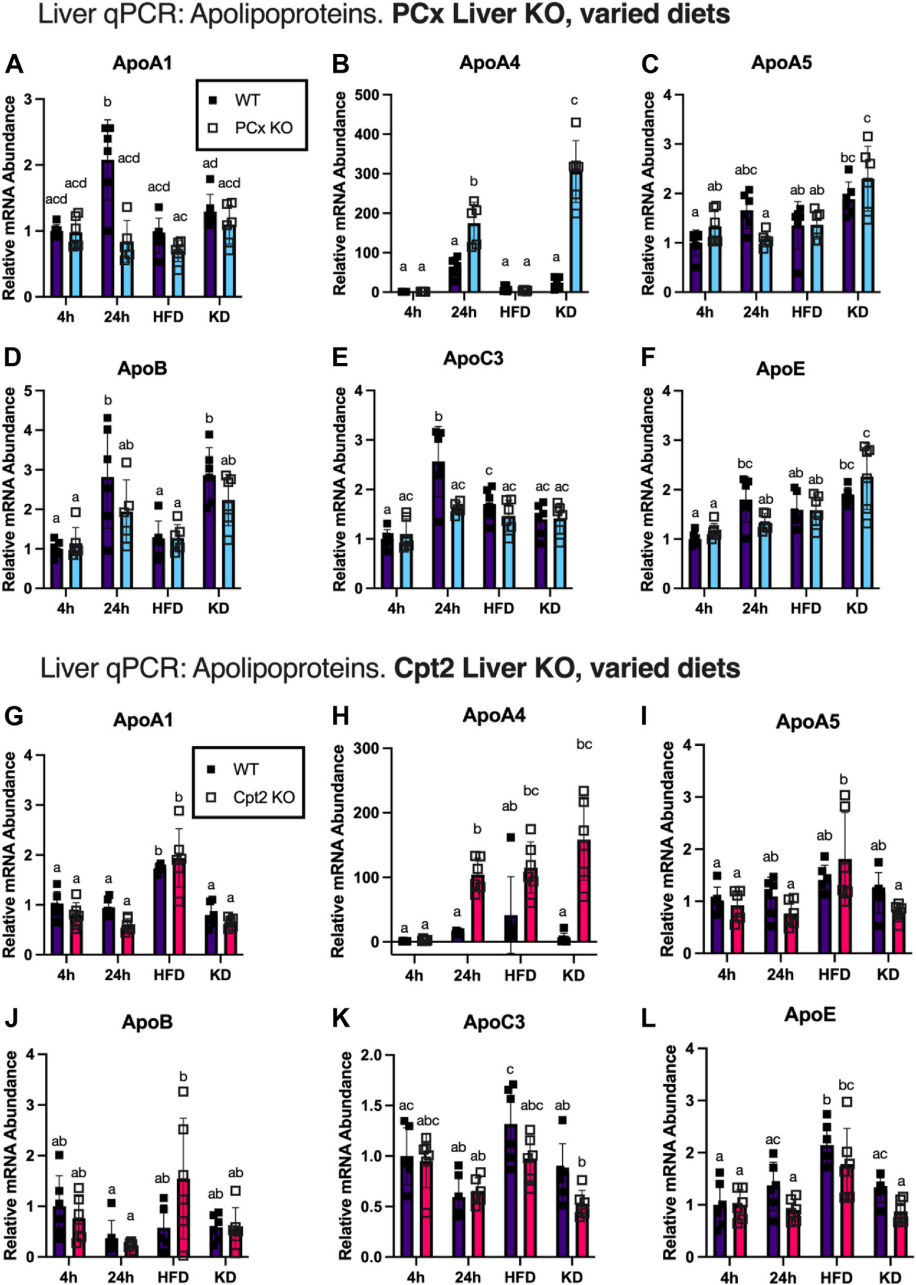
**Relative liver ApoA4 mRNA levels increase for PCx^L−/−^ and Cpt2^L−/−^ mice in different dietary states, as determined by reverse transcriptase-quantitative polymerase chain reaction.** Dietary states described as: chow diet, 4 h fast; chow diet, 24 h fast; high-fat diet, 4 h fast; and ketogenic diet, 4 h fast. *A*–*F*, liver mRNA (ApoA1, ApoA4, ApoA5, ApoB, ApoC3, and ApoE, respectively) of PCx^L f/f^ and PCx^L−/−^ mice with described dietary states, relative to chow diet 4 h fast PCx^L f/f^. *G*–*L*, liver mRNA (ApoA1, ApoA4, ApoA5, ApoB, ApoC3, and ApoE, respectively) of Cpt2^L f/f^ and Cpt2^L−/−^ mice with described dietary states, relative to chow diet 4 h fast Cpt2^L f/f^. Statistical significance was determined by two-way ANOVA with Tukey’s multiple comparisons test, conducted using GraphPad prism. Data are expressed as mean ± SD. Single-letter difference denotes *p* < 0.05 between groups. ApoA4, apolipoprotein A4; Cpt2, carnitine palmitoyltransferase 2; PCx, pyruvate carboxylase.

Although the liver is thought to play a secondary role in serum ApoA4 production (26), the considerable upregulation in our liver KO models brought us to ask whether the increased serum ApoA4 we observed originated primarily from the liver. To test this idea, we measured hepatic apolipoprotein mRNA levels through reverse transcriptase-quantitative polymerase chain reaction (RT-qPCR) in the PCx and Cpt2 liver KO mice in different dietary states (Fig. 3). ApoA4 mRNA increased dramatically from WTs in both PCx^L−/−^ livers with a 24-h fast and KD states (Fig. 3*B*). The Cpt2^L−/−^ model demonstrated a similar trend in ApoA4 upregulation; compared to 4 h-fasted chow-fed mice, ApoA4 liver mRNA in Cpt2 liver KOs increased most prominently on KD, followed by the 24-h-fasted state, and (unlike PCx^−/−^ mice) modestly in the HFD group (Fig. 3*H*).

In addition to our protein of interest ApoA4, we looked at apolipoproteins both within the gene cluster (ApoA1, C3, A5) and outside of the gene cluster (ApoB and ApoE). ApoA1 mRNA increased in 24 h-fasted PCx ff livers (Fig. 3*A*). Relative to the 4-h fast baseline, ApoB (Fig. 3*D*) and ApoC3 (Fig. 3*E*) expression were also induced in the livers of PCx^f/f^ (WT) mice with a 24 h fast; however, this effect was suppressed in PCx KOs. PCx^L−/−^ mice fed a KD also showed upregulation of ApoA5 (Fig. 3*C*) and ApoE (Fig. 3*F*), although to a much lower degree than the ApoA4 upregulation. As for the HFD PCx^L−/−^ group, there were no significant changes across any of the apolipoproteins compared to HFD WTs or chow-fed controls.

On HFD, Cpt2^L−/−^ mice and WT littermates saw an increase in liver ApoA1 (Fig. 3*G*) and ApoE (Fig. 3*L*) mRNA compared to respective chow-fed groups; however, there was no difference in these genes when comparing HFD Cpt2^L−/−^ and HFD WT mice. In contrast, ApoA5 increased in some HFD Cpt2^L−/−^ mice compared to chow-fed KOs, while WTs showed no significant difference (Fig. 3*I*). No changes in ApoA1, ApoE, ApoA5, or ApoB mRNA were observed in the 24-h-fasted or KD Cpt2^L−/−^ groups compared to chow-fed controls (Fig. 3*J*). Interestingly, while ApoA4 mRNA increases over 200-fold in Cpt2^L−/−^ KD mice, the ApoC3 mRNA for this group was reduced, albeit nonsignificantly compared to KD WTs or Cpt2^L−/−^ chow controls (Fig. 3*K*).

**Figure 4:**
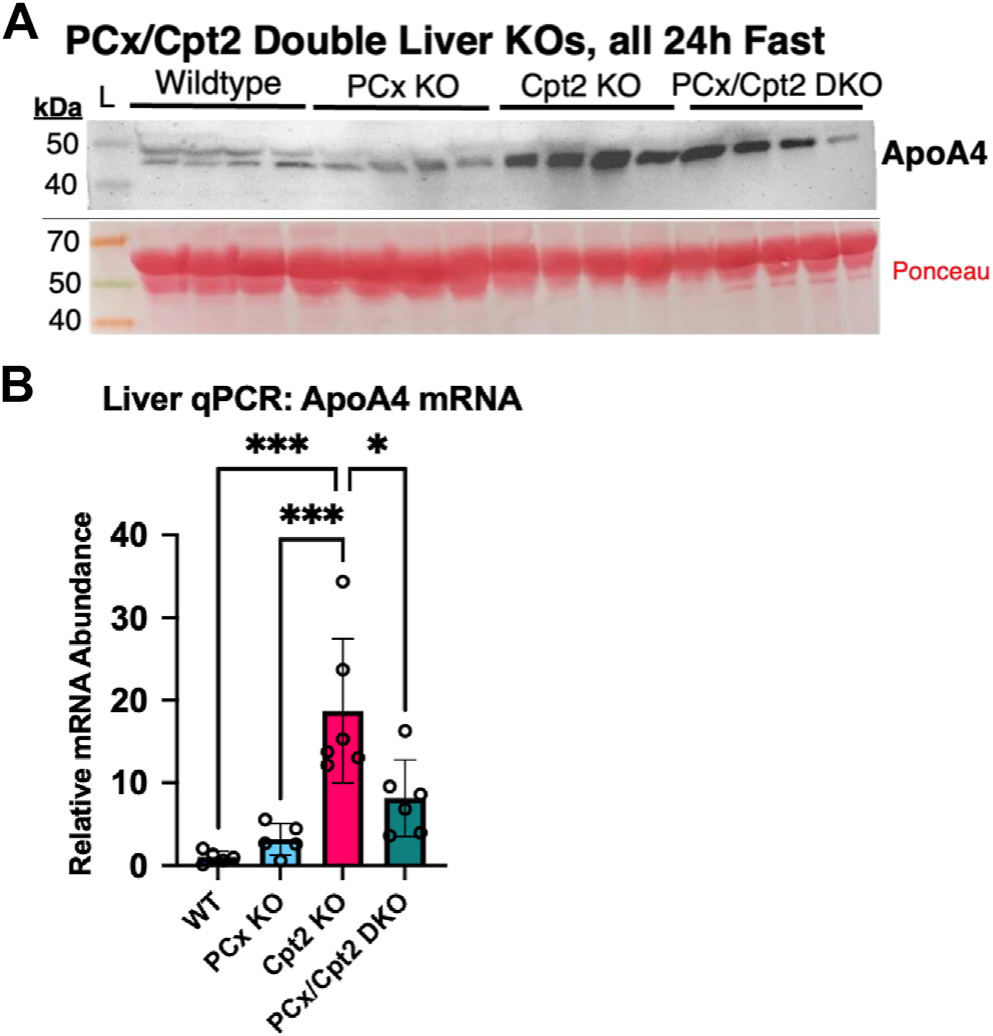
**Hepatic ApoA4 upregulation is most prominent with a 24 h fast in the absence of fatty acid oxidation.***A*, Western blots comparing serum ApoA4 levels in 24 h-fasted WT, PCx^L−/−^, Cpt2^L−/−^, and PCx/Cpt2^L−/−;−/−^ (double liver KO) mice. Ponceau-stained serum albumin bands were used as loading controls. *B*, reverse transcriptase-quantitative polymerase chain reaction comparison of ApoA4 mRNA from liver homogenate across 24 h-fasted WT (n = 5), PCx^L−/−^(n = 5), Cpt2^L−/−^ (n = 6), and PCx/Cpt2^L−/−;−/−^ mice (n = 6). In groups with n = 5, one outlier was excluded prior to statistical analysis due to aberrant Cq values for control genes (CycloA and 18s). Data are expressed as mean ± SD. ∗*p* < 0.05, ∗∗*p* < 0.01, ∗∗∗*p* < 0.001, and ∗∗∗∗*p* < 0.0001. ApoA4, apolipoprotein A4; Cpt2, carnitine palmitoyltransferase 2; PCx, pyruvate carboxylase.

As with the PCx^L−/−^ model, the upregulation of ApoA4 liver mRNA was strikingly several orders of magnitude higher than the changes in all other apolipoproteins. These data show that the combination of certain dietary and genetic models of metabolic stress produces an amplified upregulation of serum ApoA4, but not the other apolipoproteins examined. To further examine the factors that can produce a compounded upregulation of hepatic ApoA4, we proceeded utilizing a PCx/Cpt2 double liver KO model. We directly compared serum ApoA4 in 24-h-fasted WT, PCx^L−/−^, Cpt2^L−/−^, and PCx/Cpt2^L−/−;−/−^ (Fig. 4*A*). The double loss of Cpt2 did not synergistically increase ApoA4 and in fact the double KO mice experienced a slight suppression in Apoa4 mRNA (Fig. 4*B*). Overall, the high ApoA4 expression in the absence of hepatic fatty acid oxidation or PCx indicates activation of hepatic ApoA4 transcription in response to diverse metabolic distress.

### Atf3 liver mRNA increases along with ApoA4 in models of metabolic stress

**Figure 5:**
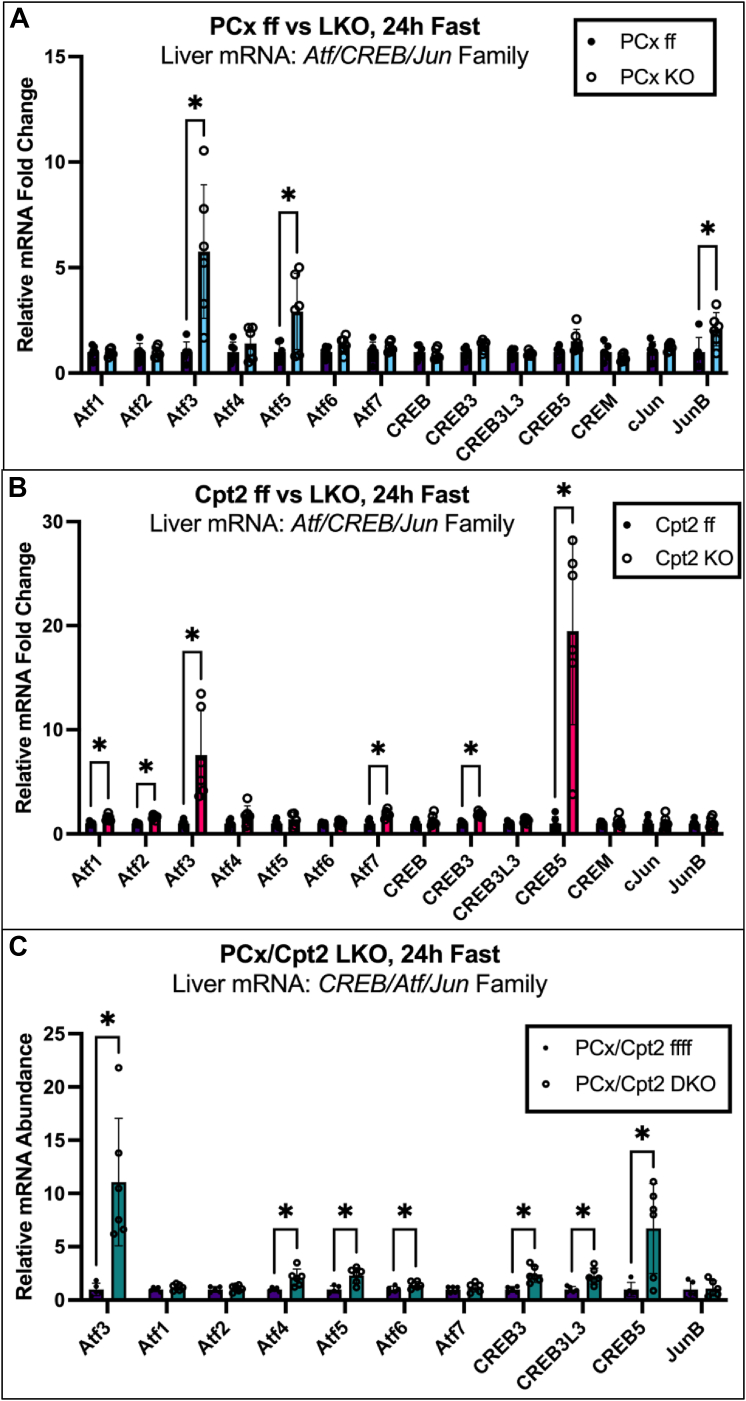
**RT-qPCR for genes in the CREB/Atf family show consistent elevation of liver Atf3 mRNA in 24 h-fasted mouse models of hepatic stress.***A*, WT and PCx^L−/−^ liver mRNA levels for CREB/Atf family members Atf 1 to 7, CREB, CREB3, CREB3L3, CREB5, and CREM, along with commonly associated proteins cJun and JunB were assessed using RT-qPCR. *B*, RT-qPCR for Atf1-7, CREB, CREB3, CREB3L3, CREB5, CREM, cJun, and JunB mRNA in WT and Cpt2^L−/−^ livers. *C*, RT-qPCR for Atf 1 to 7, CREB3, CREB3L3, CREB5, and JunB in WT and PCx/Cpt2^L−/−;−/−^ mice. Data are expressed as mean ± SD. For all graphs, Welch’s *t* tests with no correction for multiple comparisons were used to establish statistical significance (*p* < 0.05) on GraphPad prism. AFT, activating transcription factor; Cpt2, carnitine palmitoyltransferase 2; PCx, pyruvate carboxylase; RT-qPCR, reverse transcriptase-quantitative polymerase chain reaction.

To investigate the pathways leading up to ApoA4 upregulation in the liver, we measured liver mRNA levels of several members of the CREB/Atf family. We previously identified Atf3 as an upregulated gene in the livers of Cpt2 and PCx KO mice, coinciding with ApoA4 upregulation (5, 6). Two other members of this family, CREBH (encoded by gene CREB3L3) and LUMAN (CREB3) have been shown to increase ApoA4 transcription (12, 13). Because of known regulatory interactions between members of the CREB/Atf family, we also measured liver mRNA levels of a wide range of transcription factors in this family including Atf4, Atf5, CREB5, and more (Fig. 5), including Jun proteins with which CREB/Atfs often interact (11) to examine the potential influence of other relevant transcription factors.

Although various genes change significantly in hepatic expression between groups, as with ApoA4 mRNA, the only significant similarity between the PCx^L−/−^, Cpt2^L−/−^, and double KO models was an increase in Atf3 mRNA. Assessing the hepatic mRNA of 24-h-fasted PCx^L−/−^ mice also showed increased Atf5 and JunB, although to a lesser degree (Fig. 5*A*). In addition to Atf3 mRNA, Cpt2^L−/−^ livers had slightly elevated Atf1, Atf2, Atf7, and CREB3 mRNA compared to respective controls (Fig. 5*B*). CREB5 was dramatically increased in Cpt2^L−/−^ livers; this feature was also retained in the PCx/Cpt2^L−/−;−/−^ mice, although the fold change was less than half that of the Cpt2^L−/−^ mice (Fig. 5*C*). The double KOs also displayed unexpected increases in Atf4, Atf6, and CREB3L3 mRNA, in addition to the elevated Atf5 and CREB3 mRNA found in the PCx^L−/−^ and Cpt2^L−/−^ models, respectively (Fig. 5*C*).

Hepatic overexpression of human Atf3 has been documented to cause upregulation of ApoA4 (20); in contrast, the evidence linking the other upregulated genes we observed, such as Atf5 or CREB5, is lacking. There is an increase in CREB3 for the Cpt2 ^L−/−^ and double KOs, but not the PCx^L−/−^model. CREB3L3, the other confirmed ApoA4 regulator, increases in PCx/Cpt2^L−/−;−/−^ but exhibits no change in either the PCx^L−/−^ or Cpt2^L−/−^ mice. With this data, we can deduce that while some contribution is possible, neither CREB3 nor CREB3L3 are definitive drivers the hepatic ApoA4 upregulation we observe in our models. Based on its consistent elevation across disparate models, we progressed to investigating whether hepatic Atf3 would be sufficient to cause ApoA4 upregulation.

### Atf3 overexpression impacts hepatic ApoA4 upregulation in a sex-specific manner

**Figure 6:**
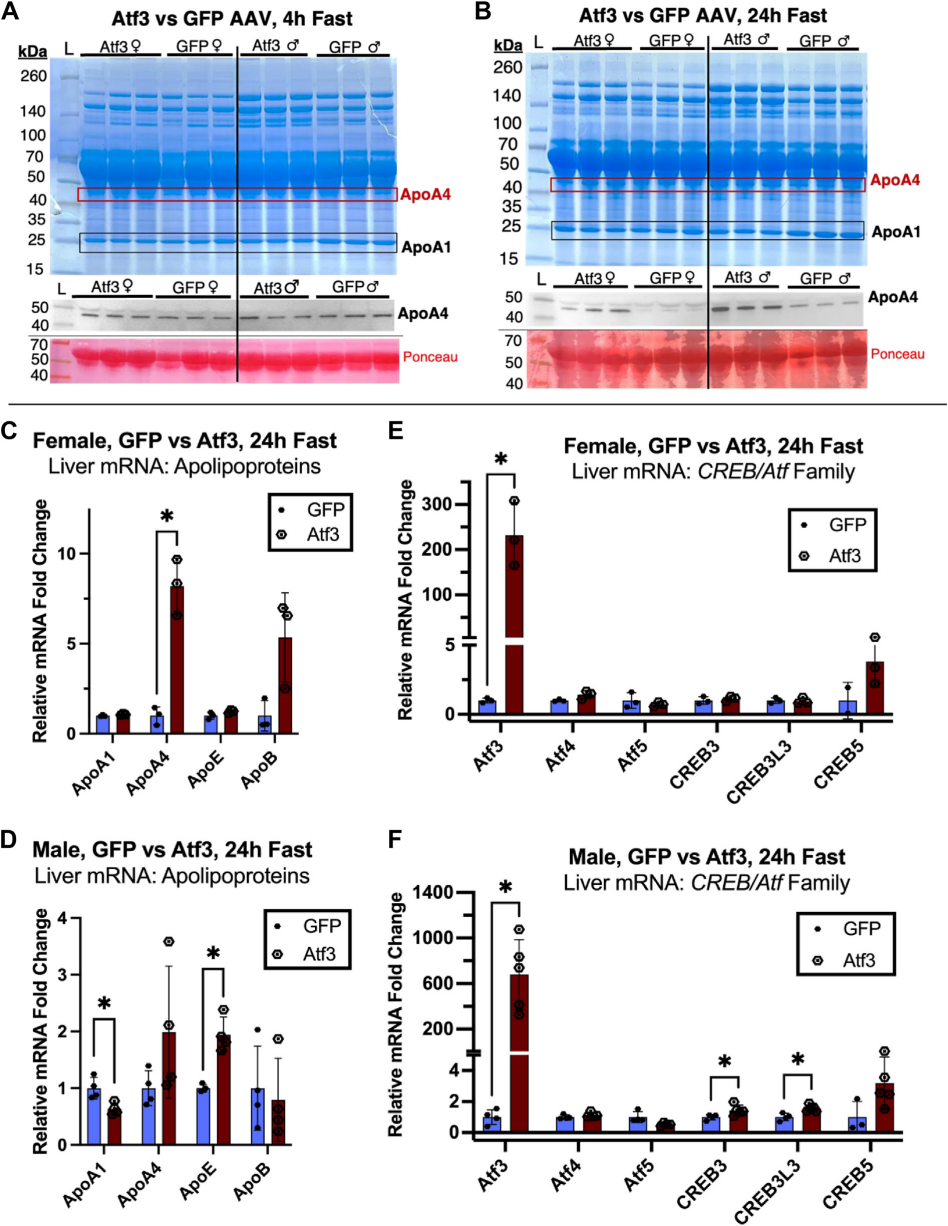
**Atf3 overexpression in WT mice significantly upregulates hepatic ApoA4 in females, but not males with a 24 h fast.***A*, coomassie-stained SDS-PAGE gels of serum proteins, followed by Western blots for ApoA4. Both male and female groups are shown for hepatic Atf3 or eGFP-overexpressing mice. Serum proteins are compared under a 4 h fast, followed by a 24 h fast replicate using the same mice (*B*) reverse transcriptase-quantitative polymerase chain reaction was used to assess hepatic mRNA abundance of apolipoproteins in 24 h-fasted female (n = 3) (*C*) or male (n = 4) (*D*) Atf3-overexpressing mice compared to GFP controls. Relevant genes in the CREB/Atf family were also assessed by reverse transcriptase-quantitative polymerase chain reaction in female (*E*) and male (*F*) groups. Data are expressed as mean ± SD and analyzed using Welch’s *t* test with no correction for multiple comparisons. All statistical tests were conducted using GraphPad prism. ApoA4, apolipoprotein A4; AFT, activating transcription factor; Cpt2, carnitine palmitoyltransferase 2; PCx, pyruvate carboxylase.

To assess the isolated effect of overexpressed Atf3 on hepatic ApoA4 expression, we sought to recapitulate the Atf3 AAV overexpression experiment by Xu *et al.*, which revealed ApoA4 upregulation through RNA-seq (21). We generated and injected 9- to 10-week-old male and female mice with ATF3 or eGFP in adeno-associated virus 8 vectors with liver-specific promoters. After 1 week, we collected serum samples from these mice and ran a Western blot to establish a baseline for circulating ApoA4 in the 4-h-fasted state (Fig. 6*A*). Two weeks postinjection, we sacrificed both groups in a 24 h-fasted state and collected their serum and livers for analysis. We confirmed hepatic Atf3 overexpression through both RT-qPCR of liver mRNA and through liver protein Western blotting for Atf3 (Supporting information S1). Western blotting of serum demonstrates a small difference in ApoA4 bands between the ATF3- and GFP-injected groups following a 24 h fast (Fig. 6*B*), compared to serum samples of the same mice from the 4 h-fasted state. We proceeded to measure liver mRNA levels for these mice to assess ApoA4 expression and to reveal any Atf3-specific transcriptional changes for other relevant genes.

With hepatic Atf3 mRNA overexpressed, ApoA4 mRNA increased significantly in females without induction of other apolipoproteins (Fig. 6*C*). This clearly indicates that Atf3 can affect ApoA4 expression; however, the nature of this transcriptional link is unclear. Complicating the matter, ApoA4 did not increase significantly in males, while mRNA for ApoE increased and ApoA1 decreased (Fig. 6*D*). Assessing for CREB/Atf mRNA in female mice showed a slight increase in Atf6 (Supporting information S1*A*) but no upregulation in the other members of the family, including CREB3 and CREB3L3, further supporting a link between hepatic Atf3 and ApoA4 expression. Interestingly, although the increase in ApoA4 mRNA was not significant in males, the liver mRNA levels of both CREB3 and CREB3L3 were slightly elevated compared to male GFP controls (Fig. 6*F*). This elevation was not present in females (Fig. 6*E*), suggesting a more complex web of sex-specific regulatory mechanisms surrounding fasting-induced upregulation of ApoA4 in Atf3 overexpression.

### ApoA4 expression is differentially impacted in female or male mice with combined Atf3 and Cpt2 liver KO

**Figure 7:**
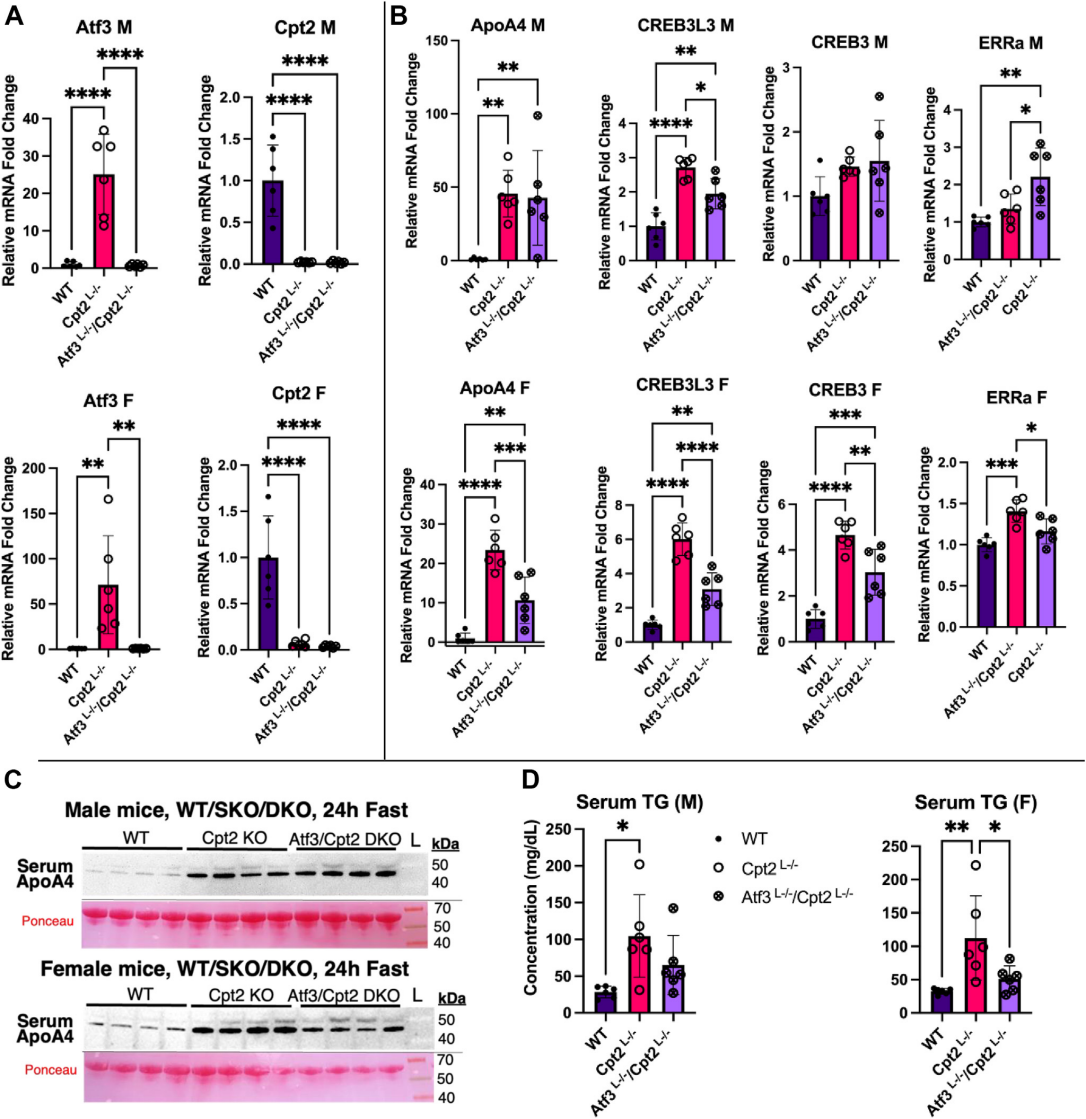
**Hepatic Atf3^L−/−^/Cpt2 ^L−/−^ double KOs demonstrate a reduced induction of hepatic ApoA4 in females, with a transcriptional profile distinct from Cpt2^L−/−^ single knockouts.***A*, validation of Atf3^L−/−^/Cpt2 ^L−/−^ double KO model using reverse transcriptase-quantitative polymerase chain reaction to show significantly reduced levels of hepatic Atf3 and Cpt2 mRNA. *B*, reverse transcriptase-quantitative polymerase chain reaction of liver mRNA for ApoA4 and its documented regulators, CREB3L3, CREB3, and ERRα. *C*, Western blots comparing serum ApoA4 levels in Atf3^L−/−^/Cpt2 ^L−/−^ males and females compared to Cpt2 ^L−/−^ mice (n = 4). Ponceau-stained serum albumin bands were used as loading controls. *D*, serum triglyceride assay comparing floxed, Cpt2^L−/−^, and Atf3^L−/−^/Cpt2^L−/−^ levels. Data analyzed using ordinary one-way ANOVA with the Brown-Forsythe test, Bartlett's test, and Bonferroni multiple comparisons test, conducted with GraphPad PRISM; n = 6. AFT, activating transcription factor; ApoA4, apolipoprotein A4; Cpt2, carnitine palmitoyltransferase 2; ERRα, estrogen-related receptor alpha; PCx, pyruvate carboxylase.

After revealing a sex-specific effect of hepatic Atf3 overexpression on ApoA4 transcription, we returned to the Cpt2^L−/−^ KO model to examine the requirement of Atf3 for the upregulation of ApoA4 within a model of metabolic stress. Because we established a dramatic induction of both hepatic Atf3 and ApoA4 mRNA in male Cpt2^L−/−^ mice, we generated a liver-specific Atf3/Cpt2 double KO mouse line to assess differences in ApoA4 regulation. We validated this model using RT-qPCR on the livers of both male and female 24 h-fasted mice (Fig. 7*A*), comparing double KOs (Atf3^L−/−^/Cpt2^L−/−)^ and single KOs (Cpt2^L−/−^) to phenotypically WT double-floxed mice (Atf3^f/f^/Cpt2^f/f^). In both male and female double KOs, the induction of hepatic Atf3 mRNA resulting from Cpt2 deficiency was successfully blocked.

Upon measuring hepatic mRNA levels of relevant genes, we again found a sex-specific difference in the impact of Atf3 expression on ApoA4 upregulation. While male DKOs had similar ApoA4 expression compared to single knockout (SKO)s, female DKOs presented with an intermediate level of hepatic ApoA4 mRNA between the WT and SKO groups (Fig. 7*B*). These data confirm a requirement of hepatic Atf3 for full induction of ApoA4 expression in female, but not male mice under hepatic stress. The cause for this difference may be partially attributed to the changes in CREB3 and CREB3L3 expression, both of which also presented intermediary mRNA levels in female DKOs. This phenomenon is seen for only CREB3L3 mRNA in male DKOs, and the magnitude of this deviation is smaller than that of the female changes. We also measured liver mRNA for estrogen-related receptor alpha (ERRα), an orphan receptor also documented to regulate ApoA4 expression by targeting the ApoA4 promoter (27). The liver mRNA for this gene did not change between male WTs and SKOs but increased in DKOs (Fig. 7*B*). Meanwhile, female SKOs showed elevated levels of ERRα, compared to slightly lower levels in both female WTs and DKOs.

To more broadly assess the transcriptional changes that occur when Atf3 is knocked out in addition to Cpt2 in a liver-specific manner, we mapped the transcriptomes of male DKOs compared to SKOs and WT mice through RNA-seq (Table S2). Gene ontology and Kyoto Encyclopedia of Genes and Genomes plots comparing Atf3^L−/−^/Cpt2^L−/−^ double KOs and Cpt2^L−/−^ single KOs show a wide array of upregulated and downregulated genes. Notably, genes for fatty acid metabolic processes and the PPAR signaling pathway are downregulated in the absence of Atf3 (Supporting informations S2, S3). Significantly changed genes relevant to lipid metabolism included Elovl7, ClpX, ApoB receptor, and others; to further assess the sex differences in the single and double KOs, we quantified the liver mRNA of these genes in males and females through RT-qPCR (Supporting information S4). These data show the requirement of ATF3 in the transcriptional response to hepatic metabolic stress.

Our final experiments sought to corroborate the sex-specific differences in hepatic ApoA4 regulation by assessing the downstream impacts of altered hepatic ApoA4 mRNA expression. Much like the Western blots in our initial screening of serum proteins, both male and female Cpt2 SKOs demonstrated high amounts of circulating ApoA4 compared to WTs (Fig. 7*C*). We observed a slight decrease in circulating ApoA4 for female DKOs compared to SKOs, a difference not seen in male DKOs. This was consistent with RT-qPCR data (Fig. 7*B*). Finally, we assessed the role of ATF3 in affecting circulating triglycerides. Whereas the serum triglyceride in male DKOs did not differ significantly from either SKO or WT groups, female DKOs demonstrated reduced serum triglyceride levels compared to SKOs (Fig. 7*F*). Although serum triglyceride levels are impacted by a wide range of factors and cannot be attributed definitively to ApoA4, these data reflect the sexual dimorphism of Atf3 and ApoA4 regulation in models of hepatic stress and may help resolve differences between males and females to metabolic dysfunction.

## Discussion

In this study, we observe the upregulation of both ApoA4 and Atf3 in disparate genetic models of metabolic enzyme deficiencies. Until now, the connection between these two genes was unexplored. Yet, existing literature reveals many functional similarities in the context of hepatic metabolism. ApoA4 deficiency in mouse and rat models aggravated diet-induced obesity and caused pronounced hepatic steatosis (11, 12), as did both global and liver-specific ablation of Atf3 (19). Furthermore, studies overexpressing either ApoA4 or Atf3 in mice increased hepatic lipolysis and fatty acid oxidation (11, 28). In models with defective fatty acid oxidation and severe hepatic steatosis like our Cpt2^L−/−^ mice, the upregulation of proteins with such protective attributes is unsurprising. This study reveals a novel connection between Atf3 and ApoA4; we propose that increased Atf3 from hepatic stress triggers ApoA4 expression through a novel liver-specific pathway, distinct from traditional ApoA4 regulation.

ApoA4 is primarily expressed in the small intestine during lipid absorption after a meal, with minor expression in the liver (26). However, our data show that catabolic states (*e.g*., 24-h fast, KD) induce an immense upregulation of liver ApoA4 in multiple mouse models with dysregulated metabolism; this suggests a novel regulatory pathway for hepatic ApoA4 in response to stress. Juxtaposing the severity of metabolic stress with the levels of ApoA4 expression in different models emphasizes the biological value of inducing ApoA4 where lipid metabolism is concerned; for example, on the HFD, Cpt2^L−/−^ mice demonstrate increased levels of ApoA4 liver mRNA when compared to chow-fed Cpt2^L−/−^ mice. This contrasts with the PCx^L−/−^ HFD group and could be attributed to the Cpt2^L−/−^ model’s restriction of lipid catabolism and a resulting lower tolerance for the HFD in Cpt2^L−/−^ mice. However, we still see hepatic ApoA4 upregulation in 24 h-fasted and KD PCx^L−/−^ mice, the latter of which demonstrated fatal metabolic stress (6).

Liver ApoA4 mRNA data are largely reflected in serum ApoA4 levels; however, there is an interesting disparity with 24 h-fasted PCx^L−/−^ mice. Although RT-qPCR shows significant ApoA4 mRNA upregulation in 24-h-fasted PCx^L−/−^ mice (Fig. 3*B*), the increase in serum protein for this group is not significant on an ApoA4 Western blot (Fig. 1, *B* and *C*). Based on the role of ApoA4, one may reason that the export of hepatic lipids into the serum is connected to the difference in ApoA4’s protein distribution between models. Although Cpt2^L−/−^ mice present with hepatic steatosis upon prolonged fasting (4), PCx^L−/−^ livers retained a normal appearence (6). Furthermore, increases in circulating triglycerides are observed in Cpt2^L−/−^ mice (4), but we found no changes in serum triglyceride for 24 h-fasted PCx^L−/−^ mice (6). These differences might be expected given that the PCx^L−/−^ model does not disturb fatty acid oxidation as the Cpt2^L−/−^ model does; perhaps it is the lack of these additional stressors that prevent a complete induction of ApoA4 activity. Where exaggerated hepatic lipid export is not needed, a secondary regulatory mechanism (*e.g.*, translational repression or increased protein turnover) could potentially prevent hepatic ApoA4 transcribed in the fasted state from reaching the serum of PCx^L−/−^ mice. Nevertheless, there is clearly an initial stress trigger for the transcriptional upregulation of ApoA4.

A study in humans revealed serum ApoA4 levels to be positively associated with age, and additional analyses showed a significant sex effect with enrichment of serum ApoA4 in males (29). Here, we revealed a sex-specific difference in the link between Atf3 and ApoA4 expression; in our examination of both Atf3-overexpressing and KO mice, evidence points to a closer correlation between changes in Atf3 and ApoA4 expression in female mice. How this interaction is mediated, however, is still unknown. Revisiting the sex differences in liver mRNA between models (Fig. 7*B*): while ERRα has no relation to the estrogen receptor or other sex hormone signaling systems in practice, there stands a possibility for its involvement in the sex-specific differences of hepatic ApoA4 regulation. However, it is worth noting the small scale of these differences in female mice compared to CREB3L3 and CREB3; the biological relevance of the ERRα mRNA variation is less clear. Comparing the liver mRNA of these genes between males and females, the sex-specific difference in ApoA4 expression could plausibly be driven by CREB3 expression in the Atf3^L−/−^/Cpt2^L−/−^ model. This inference considers the extensive crosstalk within the CREB/Atf family, linking hepatic Atf3 deficiency in females to the obstructed induction of CREB3 on a Cpt2^L−/−^ background.

Atf3 is rapidly induced in several tissues in response to stress signals, and as an immediate-early gene, it is involved in various signaling pathways (30). This wide range of activity could be attributed to Atf3’s many potential sites for posttranslational modification (30), or that it can act as a transcriptional activator or repressor depending on its binding partner. For example, Atf3 homodimers repress transcription, and heterodimers with partners such as c-Jun and other CREB/Atfs can activate its target promoters (16, 31). Even promoters without Atf3 binding sites could be activated by Atf3 expression *via* mechanisms like sequestering suppressive factors (31).

ATF3-AAV–injected mice vastly overexpressed hepatic Atf3 mRNA compared to GFP controls, although to a much higher magnitude in males than females. Given the documented suppressive effect of Atf3 dimers, it stands to reason that the excessive upregulation of Atf3 in male mice could result in a decreased ApoA4 response *via* the transcriptional repression of intermediate actors; however, we did not observe this for at least two known ApoA4 regulators (CREBH, LUMAN). Extending beyond transcription, it is also possible that high levels of Atf3 protein could sequester and thus alter the activity of other ApoA4 transcriptional regulators, independent of mRNA or protein levels.

Conflicting research exists attributing both protective and detrimental roles to Atf3 in hepatic metabolism. Studies using the TTR-ATF3 vector for Atf3 overexpression, for example, report repressed gluconeogenesis and symptoms of liver dysfunction in transgenic mice (18, 19). Because this vector also targets the pancreas, the overexpressed Atf3 could also play a role in stress-induced beta cell apoptosis (32); this impact of Atf3 on the pancreas could subsequently contribute to the issues in hepatic metabolism associated with persistent high Atf3 expression in ZDF rats and in humans with nonalchoholic fatty liver disease and/or diabetes (16). Notwithstanding the organ-specific effects of Atf3, recent studies specifically focused on liver metabolism still report divergent roles of Atf3 in hepatic stellate and macrophage cells. Overexpression of Atf3 in isolated LX-2 cells by Li *et al.* promoted increased stellate cell migration, and *in vivo* Atf3 knockdown in *Schistosoma japonicum*–infected mice reduced liver fibrogenesis (33). On the other hand, Hu *et al.* report that Atf3 *overexpression* in hepatic macrophages decreased fibrogenesis in addition to decreasing hepatic lipid accumulation and increasing genes involved with lipolysis and fatty acid oxidation (34). These results reinforce the similarities between the metabolic effects of liver Atf3 and ApoA4 and indicate precision in the hepatic regulation of these proteins at the cellular level.

In conclusion, this study identifies a distinct role of hepatic ApoA4 in multiple models of metabolic stress and reveals a novel connection between ApoA4 and Atf3 with sex-specific differences. The multifaceted roles of both proteins within and beyond liver metabolism underscore the merit of continued investigation into the link between Atf3 and ApoA4.

## Experimental procedures

### Mouse models

Current and previous lab members developed the C57BL6 mouse lines from which the samples in the protein screen were acquired; to generate liver KOs, floxed mice were bred to Albumin-Cre mice previously described (4, 5, 6, 15). Here, we generated Atf3-floxed mice. C57BL/6N-Atf3<tm2a (EUCOMM)WTs from the European Conditional Mouse Mutagenesis consortium was first bred to Flpe germline deleter mice (Jax #5705) to obtain mice with a floxed Atf3. Genotyping primers used to confirm floxed genes and detect Cre recombinase alleles are listed in Table S1. Mice were housed in ventilated racks with a 14 h-light/10 h dark cycle, and fed a standard chow diet (2018SX, Envigo Teklad Diets), KD (AIN76A-Modified, High Fat, Paste; F3666, Bio-Serv), or a 60% HFD (#D12492, Research Diets) as previously described (5, 6). Prior to sacrifice, mice were fasted for either 4 h (11 AM–3 PM) or 24 h (3 PM–3 PM) with water provided *ad libitum*. To overexpress hAtf3 in WT mice, we retro-orbitally injected $2\times 10^{11}$ genome copies of an adeno-associated virus vector 8 containing either TBG-eGFP (SKU: VB1743, Vector BioLabs) or Flag-ATF3 driven under the Albumin promoter (Vector BioLabs). One-week post-AAV injection, we collected blood from the tail vein in the 4-h-fasted state. We sacrificed AAV-treated mice in the 24 h-fasted state 2 weeks postinjection. All procedures were performed in accordance with the NIH’s Guide for the Care and Use of Laboratory Animals and under the approval of the Johns Hopkins School of Medicine Animal Care and Use Committee.

### Coomassie staining

Serum was collected and spun at 10,000 RCF for 10 m. Supernatant was collected and stored at −80 °C until use. Samples were prepared using 4x Laemmli sample buffer (Bio-Rad) according to manufacturer protocol. One microliter of serum per well was loaded onto 4 to 15% Criterion TGX Precast Midi Protein Gels (Bio-Rad) and run at 200 V for approximately 40 m. At this point, gels were extracted from the casing. The gels were washed in deinonized water and stained using SimplyBlue SafeStain (Invitrogen) according to manufacturer’s High-Sensitivity Protocol. Imaging was conducted on an Alpha Innotech Multi ImageIII instrument.

### Western blot

For liver protein Western blotting, proteins were extracted using radioimmunoprecipitation assay lysis buffer with cOmplete protease inhibitor (#11836170001, Roche) and PHOSSTOP phosphatase inhibitor (#4906847001, Roche), and tissues were fully lysed through bead homogenization. Concentrations of liver protein homogenates were assessed and adjusted to 50 ul/well following Pierce bicinchoninic acid assay Protein Assay (#23227, Thermo Fisher Scientific). SDS-PAGE gels were run according to the Coomassie stain procedure up to the gel extraction step. Proteins transfer from SDS-PAGE gels to polyvinylidene difuoride membranes facilitated using the TurboBlot Transfer system (Bio-Rad). Ponceau S staining (#59803, Cell Signaling) for 10 min was used to verify consistent protein transfer. Following ponceau stain, membranes were washed thrice in Tris-buffered saline with Tween-20 (TBST) for 5 min each, and blocked with 5% nonfat milk in TBST for 2 h at room temperature. After another 3 × 5 m TBST washes, blots were incubated with primary antibodies in 3% bovine serum albumin overnight at 4 °C; ApoA4 (#AF8125, R&D systems), Atf3 (#33593, Cell Signaling), and HSC70(sc-7298, Santa Cruz). Blots were incubated with secondary antibodies in 5% milk for 2 h at room temperature: ApoA4 (anti-sheep horseradish peroxidase, #A3415, Sigma), Atf3 (anti-rabbit horseradish peroxidase, #7074S, Cell Signaling), HSC70 (Anti-Mouse Cy3, M30010, Thermo Fisher Scientific). Antibodies were previously validated (35). Amersham ECL Select (Cytiva) was used for imaging on an Alpha Innotech Multi ImageIII instrument. The Alpha Innotech AlphaView software was used for quantitation of Western blot bands, normalizing to local background to account for variable exposure. Further data analysis was conducted using Excel and GraphPad prism (https://www.graphpad.com).

### Reverse transcription-quantitative PCR

Tissue samples were collected, flash-frozen, and stored at −80 °C. Tissues were homogenized in Trizol reagent (Invitrogen). Total RNA was extracted by preparation according to Qiagen protocol, followed by the Qiagen RNeasy mini kit. RNA quality was assessed by Nanodrop.

Complemenatry DNA was synthesized from 2 ug RNA using the High-Capacity complemenatry DNA Reverse Transcription Kit (Thermo Fisher Scientific). Quantitative PCR was performed using SsoAdvanced Universal SYBR Green Supermix (Bio-Rad) following manufacturer recommendations. *Cyclophilin* A and *18s* were used to normalize Cq values; additional primers used are listed in Table S1. RT-qPCR data analysis was performed on Excel and GraphPad PRISM.

### Triglyceride assays

Serum samples were collected, flash-frozen, and stored at −80 °C until use. Serum triglycerides were measured using the Infinity Triglycerides Stable Liquid Reagent kit (Thermo Fisher Scientific, TR-22421) and a glycerol standard solution (MilliporeSigma, G7793). Data were collected using an Agilent Biotek Gen5 microplate reader and imagine software (ver. 3.13; www.agilent.com) and processed using GraphPad PRISM.

## Data availability

GEO accession: GSE288486.

## Supporting information

This article contains supporting information.

## Conflict of interest

The authors declare that they have no conflicts of interest with the contents of this article.

## Supporting information

Supplemetary Table 1
