# Supplementary material for: Activating transcription factor 3 regulates hepatic apolipoprotein A4 upon metabolic stress
Source: J Biol Chem. 2025 Mar 28;301(5):108468. doi: 10.1016/j.jbc.2025.108468 (PMC12059330; doi:10.1016/j.jbc.2025.108468)
Supplement: Supplemetary Table 1 [file mmc1.pdf]

Supporting Information Table 1. Genotyping and RT-qPCR primer sequences for all genes.

Genotyping Primers

Gene Name	Abbreviation	Forward primer	Reverse primer
Cre Recombinase	Cre	CCAGCTAAACATGCTTCATCG	CTAACCAGCGTTTTCGTTCTG
Carnitine palmitoyltransferase 2	Cpt2	CAACTCGTATACCCAAACCCAGTC	GTTCCCATCTTGATCGAGGACATC
Activating Transcription Factor 3	Atf3	CAC TGC AGC TGT CTT TAG	ATC AGT GAA CTG ATG GCG

RT-qPCR Primers

Gene Name	Abbreviation	Forward primer	Reverse primer
Cyclophilin A	CycloA	TCCGACTGTGGACAGCTCTA	ATTGCGAGCAGATGGGGTAG
18s	18S	GCAATTATTCCCCATGAACG	GGCCTCACTAAACCATCCAA
Apolipoprotein A4	ApoA4	CCA GCT AAG CAA CAA TGC C	TGG AAG AGG GTA CTG AGC TGC
Apolipoprotein A1	ApoA1	AGA AGA GCT GGA CAC CCA GA	CAC CAC AGC TTT CAT CCT GA
Apolipoprotein A5	ApoA5	GAC ACC TAC CTG CAG ATT GCT G	TTC CCA CAG GTC GTC CAG TCG
Apolipoprotein B	ApoB	CAA GCA CCT CCG AAA GTA	CAC GGT ATC CAG GAA CAA
Apolipoprotein C3	ApoC3	GCA TCT GCC CGA GCT GAA GAG	CTG AAG TGA TTG TCC ATC CAG C
Apolipoprotein E	ApoE	CTC CCA AGT CAC ACA AGA ACT G	CCA GCT CCT TTT TGT AAG CCT TT
Activating Transcription Factor 3	Atf3 qPCR	CCC TGA AGA AGA TGA GAG	TCA GCA TTC ACA CTC TCC
Activating Transcription Factor 1	Atf1	GATTCCCACAAGAGTAACACGAC	CCTATGCTGTCAGATGAGTCCT
Activating Transcription Factor 2	Atf2	CCGTTGCTATTCCTGCATCAA	TTGCTTCTGACTGGACTGGTT
Activating Transcription Factor 4	Atf4	ATG GCG CTC TTC ACG AAA TC	ACT GGT CGA AGG GGT CAT CAA
Activating Transcription Factor 5	Atf5	CTG GCT CCC TAT GAG GTC CTT G	GAG CTG TGA AAT CAA CTC GCT CAG
Activating Transcription Factor 6 (Aka Crebl1)	Atf6	TGGAGCAGGATGTCCCGTT	CTGTGGAAAGATGTGAGGACTC
Activating Transcription Factor 7	Atf7	ATGGGAGACGACAGACCGTT	GGCGTTTGATCTGCAATGATGA
cAMP Response Element Binding Protein (CREB) 1	CREB	AGCAGCTCATGCAACATCATC	AGTCCTTACAGGAAGACTGAACT
CREBH/CREB3L3 (mouse)	CREB3L3	CCTGTTTGATCGGCAGGAC	CGGGGGACCATAATGGAGA
CREB3/LZIP/LUMAN (mouse)	CREB3	AAGGCTCCGCTGGACTTAGA	TGTGGAAGGGAGTAGTTGTGA
CREB 5	CREB5	AGGATCTTCTGCCGTCTTGAT	GCGCAGCCTTCAGTCTCAT
cAMP response element modulator	CREM	TTGCCCCAAGTCACATGGC	ACTGCGACTCGACTCTCAAGA
c-Jun	c-Jun	GTCCTCCATAAATGCCTGTTCC	GATGCAACCCACTGACCAGAT
JunB	JunB	TCACGACGACTCTTACGCAG	CCTTGAGACCCCGATAGGGA
Carnitine palmitoyltransferase 2	Cpt2	CAACTCGTATACCCAAACCCAGTC	GTTCCCATCTTGATCGAGGACATC
ELOVL family member 7	Elovl7	CATCGAGGACTGTGCGTTTTT	CCAGGATGATGGTTTGTGGCA
Matrix metallopeptidase 27	Mmp27	GCCTTGATGTTCCCCAATTACA	GCTTGGTGGTTACCTTAGGTGG
Apolipoprotein B receptor	Apobr	TGGGCTACATCAGGCTTTGAG	CTCTCCTACAACCTTCCCCTC
Caseinolytic peptidase X (E.coli), transcript variant 1	Clpx	TCCGCGCAGAGAGGTATTTC	GGCAAAGTATGCTGGTGTTTCT
Glycoprotein nmb	Gpnmb	AGAAATGGAGCTTTGTCTACGTC	CTTCGAGATGGGAATGTATGCC
