# Supplementary material for: Activating transcription factor 3 regulates hepatic apolipoprotein A4 upon metabolic stress
Source: J Biol Chem. 2025 Mar 28;301(5):108468. doi: 10.1016/j.jbc.2025.108468 (PMC12059330; doi:10.1016/j.jbc.2025.108468)

SUPPORTING INFORMATION FIGURE LEGENDS

Supporting Information 1. Liver mRNA and protein in Atf3-overexpressing mice. RT-qPCR was used to assess mRNA abundance of Atf1-7, CREB, CREB3, CREB3L3, CREB5, CREM, c-Jun, and JunB in 24-hour fasted female ***(SI1a)*** or male ***(SI1b)*** mice injected with ATF3-AAV or eGFP-AAV. Statistical significance determined by multiple unpaired t tests with Welch's correction and no correction for multiple comparisons (GraphPad PRISM). Data are expressed as mean ±SD. *p<0.05. ***(SI1c)*** Male mice were assessed for increased liver Atf3 protein by Western Blot followed to confirm excess hepatic Atf3 protein with HSC70 loading control. (n=4)

Supporting Information 2. Gene Ontology (GO) plots comparing the RNA abundance of different gene groups in Atf3$^{L-/-}$/Cpt2$^{L-/-}$ and Cpt2$^{L-/-}$ livers.

Supporting Information 3. KEGG plots comparing RNAseq data from Atf3$^{L-/-}$/Cpt2$^{L-/-}$ and Cpt2$^{L-/-}$ livers.

Supporting Information 4. Liver mRNA for Atf3$^{L-/-}$/Cpt2$^{L-/-}$ liver double knockouts compared to floxed and single knockout groups. RT-qPCR was used to assess mRNA abundance of Elovl7, Clpx, Gpnmb, CREB5, Apobr, and ApoB ***(SI4)*** in 24-hour fasted floxed, Cpt2$^{L-/-}$, and Atf3$^{L-/-}$/Cpt2$^{L-/-}$ mice. Statistics determined by ordinary one-way ANOVA with the Brown-Forsythe test, Bartlett's test, and Bonferroni Multiple Comparisons test, conducted with GraphPad PRISM; n=6.

Supporting Information 1

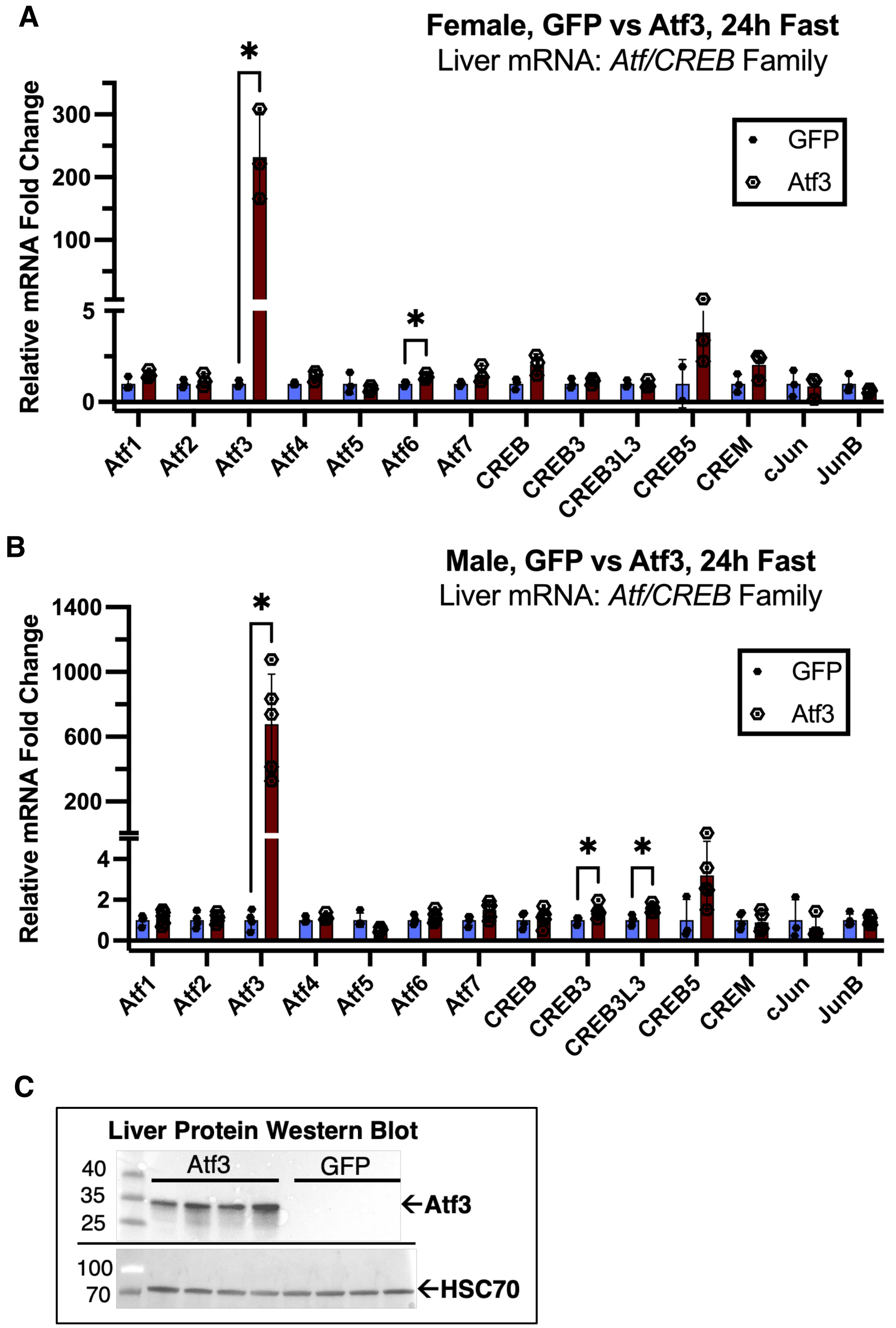

Supporting Information 2

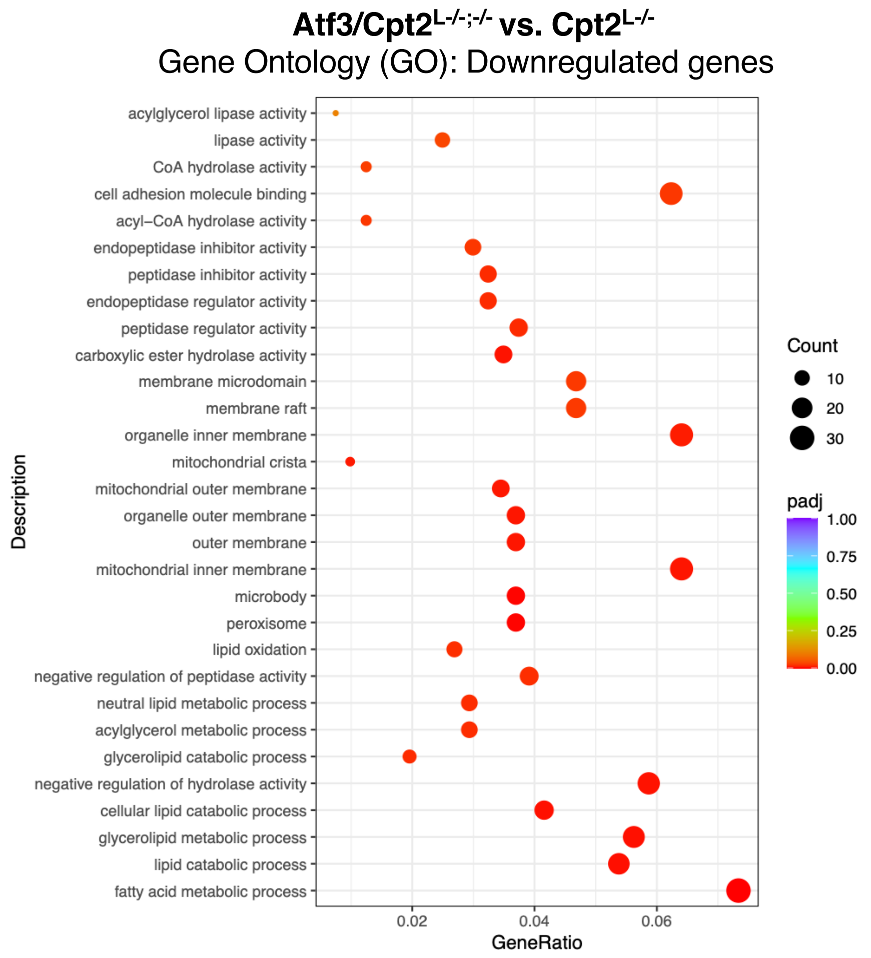

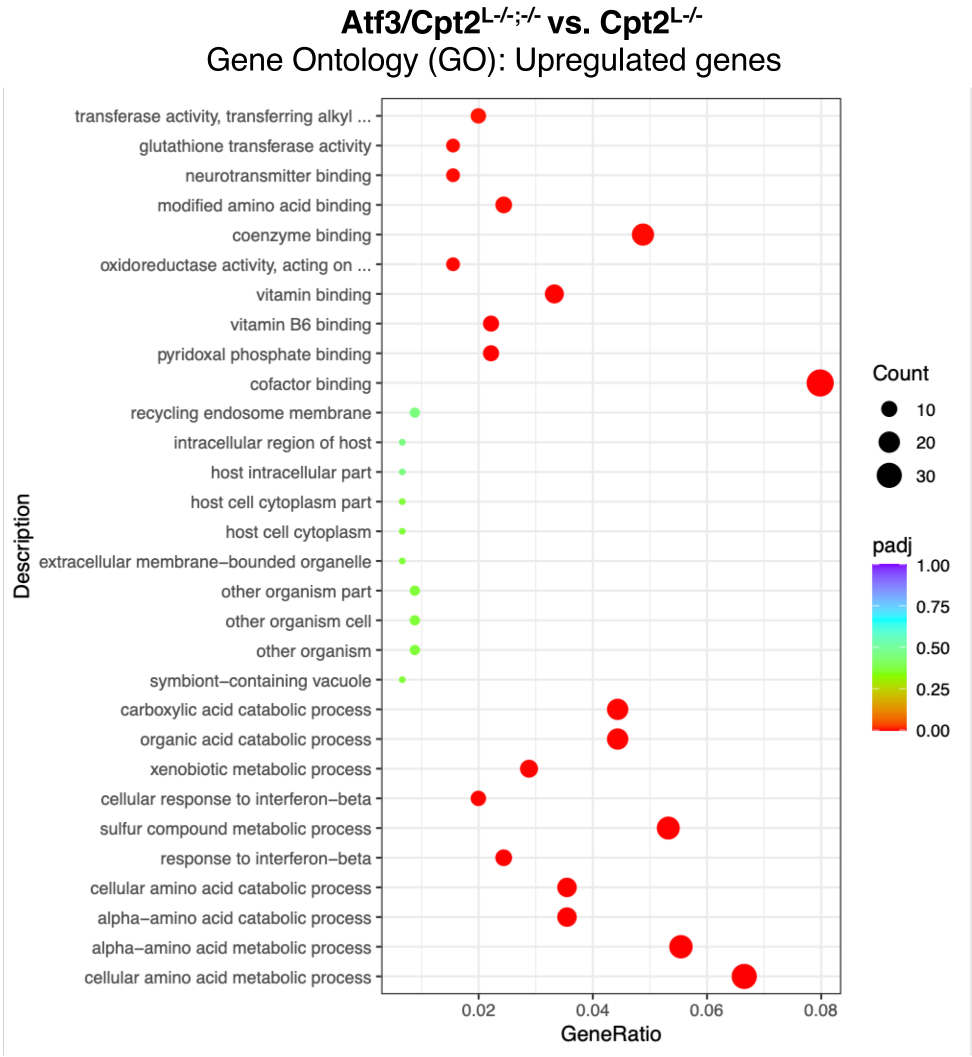

Atf3/Cpt2$^{L-/-;-/-}$ vs. Cpt2$^{L-/-}$
KEGG: Downregulated genes

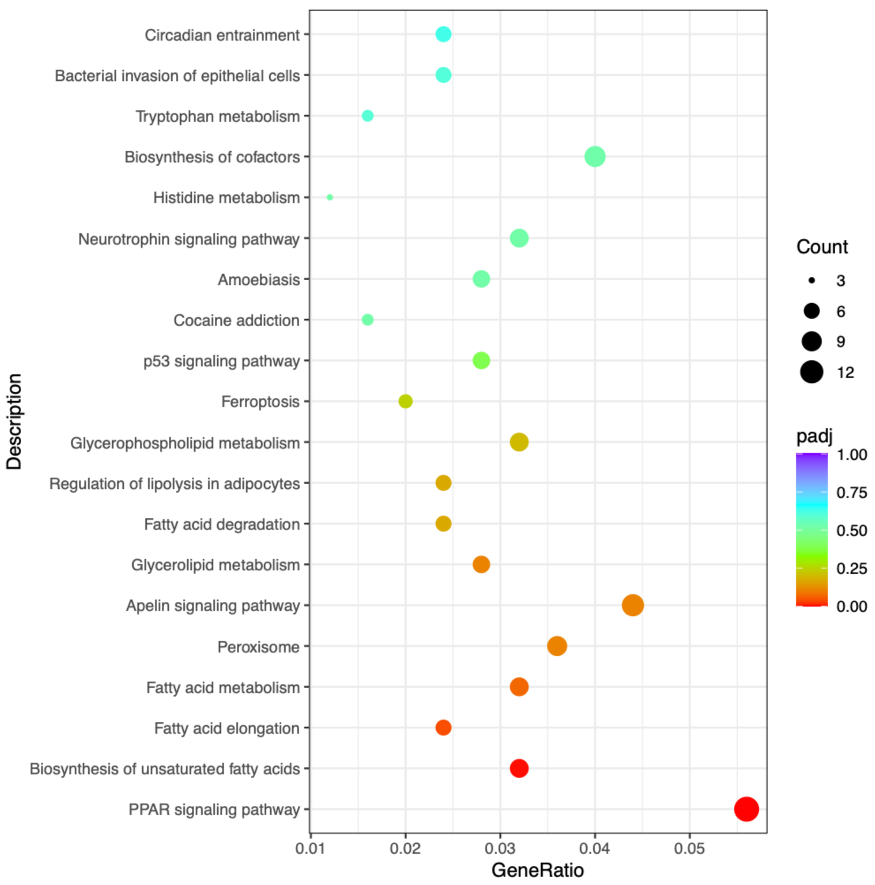

Atf3/Cpt2$^{L-/-;-/-}$ vs. Cpt2$^{L-/-}$
KEGG: Upregulated genes

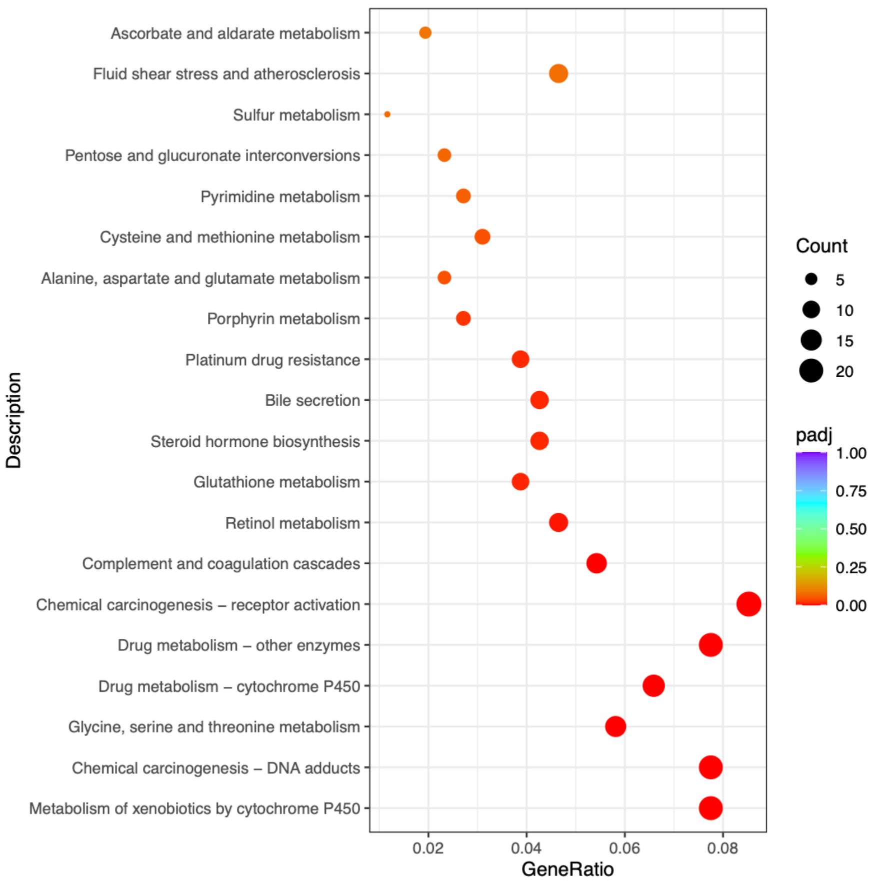

Supporting Information 4

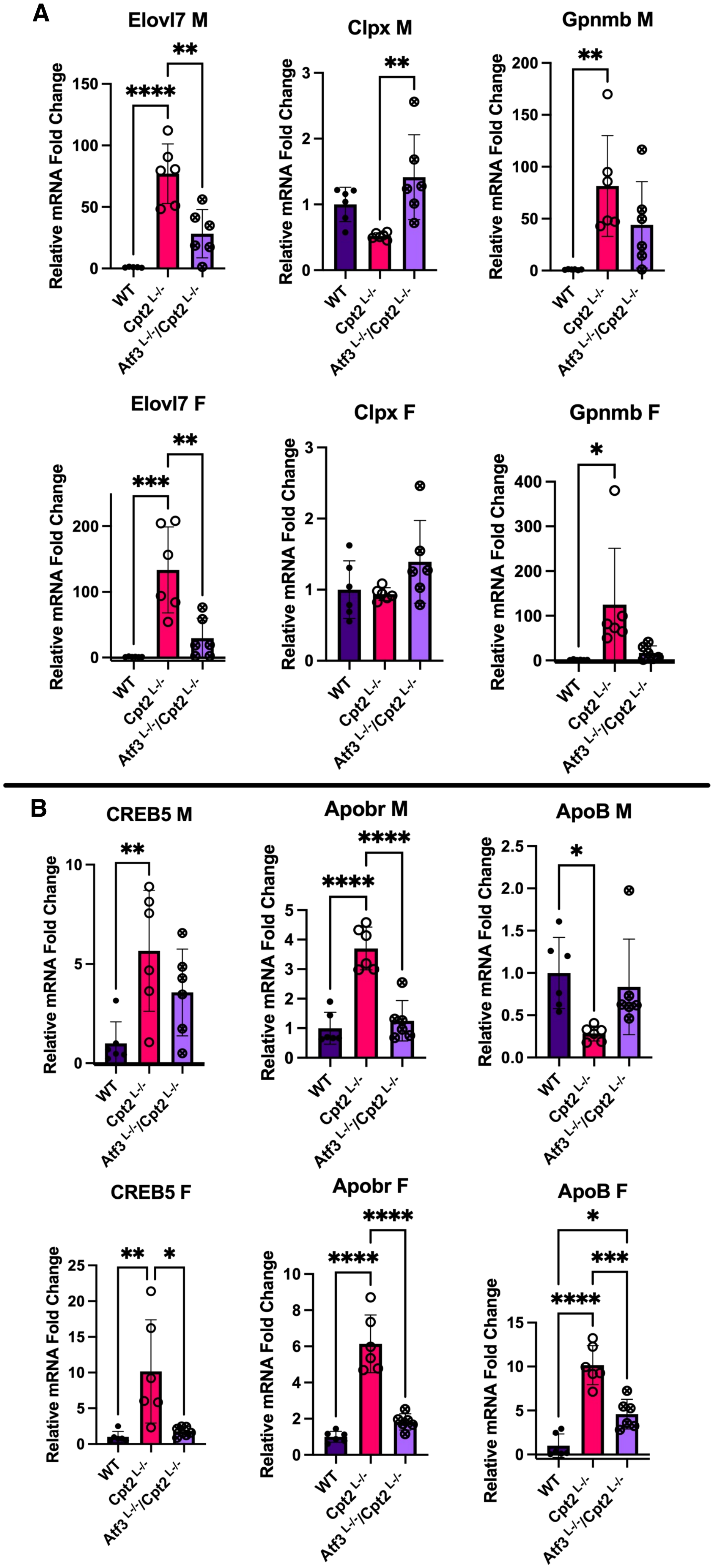

Supplement: Supplementary Figs [file mmc2.pdf]
